# *Monomorium* ant is a carrier for pathogenic and potentially pathogenic bacteria

**DOI:** 10.1186/s13104-019-4266-4

**Published:** 2019-04-16

**Authors:** Jenan S. Alharbi, Qaderya Alawadhi, Simon R. Leather

**Affiliations:** 1 Science Department, College of Basic Education, Public Authority for Applied Education and Training (PAAET) , Alardyia, PO Box: 23167, Safat, Kuwait; 2 Crop & Environment Sciences, Harper Adams University, Edgmond, Newport, TF10 8NB UK

**Keywords:** Ant pathogenicity, Bacterial types, Risk on health, House hygiene, Infection by *Salmonella*, Pathogen carrier

## Abstract

**Objectives:**

Household ants are regarded as a major household pest and their close association with microorganisms and people means that they may constitute a disease risk. Our study is the first to provide information on the pathogenicity of *Monomorium* spp. a common insect in Kuwait by quantifying and identifying the exoskeleton bacterial burden. Samples of *Monomorium* were collected in June from indoor and outdoor sites of 30 houses located in two residential districts.

**Results:**

The study identified a total of 16 different species of Gram-negative bacteria of which the indoor isolates were 75% greater in species count than the outdoor samples. Indoor isolates identified from both districts were more frequent than the outdoors and similar trends were obtained for a single district. Outdoor ant samples on the other hand carried a high percentage of bacteria but with less diversity in both districts. There was a significant variability in bacterial species in relation to sample sources, indoor and outdoor, and discrete geographical location. The presence of a high percentage of pathogenic and potentially pathogenic bacteria indoor poses a great threat to domestic households, which would be further exacerbated in places with poor standards of hygiene.

## Introduction

Many insects are important disease vectors facilitating the transmission of various microbes such as viruses, bacteria, fungi and nematodes [1]. Among those insects are some social ones such as ants, which can transmit diseases and cause allergic reactions [2, 3]. As social insects, ants generally occur in large colonies and often enter buildings where inadequate control practices and improper storage of food that might aggravate their infestation [4, 5].

*Monomorium* ants pose major public-health challenges due to their potential to spread infectious diseases and their resistance to multiple types of infection [6]. A number of ant species have become pests in many countries worldwide, invading households and residential units and posing significant health hazards. For instance, the sting of the black ant, *Pachycondyla sennaarensis*, causes allergic reactions ranging from mild to anaphylactic [7]. In the United States, the fire ant *Solenopsis* spp. and carpenter ant *Camponotus* spp. are considered serious pests in almost 6% of households, and *Monomorium pharaonis* L. and *Monomorium minimum* and other species pose threats to 13% of residential units [8–10] and may cause food poisoning that represent a danger to patients in hospital environments [11]. Nevertheless, bacterial diseases transmitted via food present a public health problem all over the world leads to high mortality and morbidity rates, [12].

Ants and bacterial associations have been detected in many hospitals, raising concerns about the possible role of ants as a disease vectors and microbial dispersal [13–15]. Several studies conducted in hospitals have shown a mutualistic relationship between ants and the presence of bacteria found on the ants’ exoskeleton [16]. A study performed in Brazilian hospitals showed that the ants *Paratrechina* spp. and *Monomorium floricola* have high capacity of reproduction and colonization rates [17]. Bacterial isolates from those ants revealed 68.8% of *Bacillus* spp. and *Listeria* spp., 14.7% of *Pseudomonas aeruginosa* and *Klebsiella* spp., and 16.4% of *Streptococcus* spp. and *Staphylococcus aureus*. These bacteria are responsible for hospital-acquired (nosocomial) infections and associated with potentially serious medical problems [16, 17]. Microbiological analysis of the cuticle of the genus *Pheidole* (subfamily Myrmicinae) revealed (90%) of *Staphyloccocus* spp. with low frequency of *Klebsiella* spp. (2%), *Enterococcus* spp. (2%) and *Vibrio cholera (*2%) [11].

*Monomorium* spp. is a common insect frequently found indoors and outdoors in residential and non-residential buildings in Kuwait. The emergence of antimicrobial resistance among the most important pathogenic bacteria coupled with the increase in ant colonies could lead to radical health consequences, ineffective infestation control measures [18] and eventually, high economic and social costs [19]. It is thus necessary to evaluate its potential pathogenicity for raising awareness of its dangers. Therefore, the aim of this study was to determine and compare the concentration of air borne bacteria conveyed by the ant *Monomorium* from indoor and outdoor areas of two different districts. To the best of our knowledge no such study has been attempted on *Monomorium* in Kuwait.

## Main text

### Materials and methods

Using gloves, worker ants of *Monomorium* were collected from outdoor (backyard) and indoor (living room) sites of 30 houses in Al-Rowda (44.1 °C and RH 52%) and Bayan districts (41.3 °C and RH 74%) during the day. In the laboratory, the ants were randomly picked from containers using forceps and transferred into sterile dilution bottles containing peptone water. Ten milliliters of sterile normal saline (0.9%) was added to each test tube containing 30 ants and the tubes were thoroughly shaken for 2 min to isolate microorganisms from the external surface. Serially diluted (10^−1^ to 10^−7^) 0.1 ml aliquots were then separately inoculated onto nutrient agar plates and incubated overnight at 37 °C. Bacterial colonies were initially identified by morphological appearance, microscopic examination using staining techniques, and identified further by biochemical tests and were characterized on an API 20E (BioMerieux, France). The overall load of bacteria carried by each ant was quantified and expressed in colony- forming units (CFUs). Gram’s staining was carried out to find the reactions of the bacterial isolates following Muhammad et al. [20].

### Results

The present study was conducted to isolate and identify bacteria existed on the workers integument collected from indoor and outdoor of residences located in Al-Rowda and Bayan districts. Bacterial species prevalence rates (%) are presented in Table 1. Indoor bacterial isolates were found to be 75% higher than outdoors irrespective of the district. The study isolated a total of 16 different species of Gram-negative bacteria and of these, 12 species were from indoor samples and the rest outdoors. The results revealed variability in the bacterial burden of ants gathered from inside and outside houses. Most of the bacterial isolates were pathogenic to potentially pathogenic. The highest percentages of occurrence of bacterial species indoors were *Shigella sonnei* (30%) at Al-Rowda, followed by *Acinetobacter lwoff* (20%). In Bayan, the highest bacterial isolates were *Serratia marcescens* (30%) and *Salmonella choleraesius* (25%). Outdoor samples varied by having the least quantity of isolates, four of which were not found among indoor isolates; *Proticus mirabilis* and *Pseudomonas luteola* in Al-Rowda and *Hafnia alvei* and *Klebsiella ozaenae* in Bayan.

**Table 1:** The means of numbers of CFU/0.1 ml aliquots obtained in three different replicates using 30 ants

Sample ID	Number of CFU/0.1 ml aliquots (mean ± SE)	Bacterial identification (API 20 E)	%
Indoor 1 (Al-Rowda)	7.0 ± 0.2 × 10^5^	*Shigella sonnei*	24
	5.0 ± 0.1 × 10^5^	*Acinetobacter lwoffii*	20
	4.0 ± 0.2 × 10^5^	*Morganella morganii*	15
	3.0 ± 0.1 × 10^5^	*Serratia marcescens*	11
	2.0 ± 0.0 × 10^5^	*Escherichia coli*	11
	2.0 ± 0.0 × 10^5^	*Yersinia enterocolitica*	9
	1.0 ± 0.0 × 10^5^	*Acinetobacter baumannii*	6
	1.0 ± 0.0 × 10^5^	*Salmonella choleraesuis*	4
Indoor 2 (Bayan)	8.0 ± 0.2 × 10^5^	*Serratia marcescens*	30
	6.0 ± 0.2 × 10^5^	*Salmonella choleraesuis*	25
	4.0 ± 0.1 × 10^5^	*Escherichia coli*	20
	2.0 ± 0.1 × 10^5^	*Pantoea agglomerans*	15
	1.0 ± 0.0 × 10^5^	*Yersinia enterocolitica*	5
	1.0 ± 0.0 × 10^5^	*Acinetobacter baumannii*	5
Outdoor 1 (Al-Rowda)	8.0 ± 0.2 × 10^5^	*Proticus mirabilis*	40
	6.0 ± 0.2 × 10^5^	*Serratia marcescens*	25
	4.0 ± 0.2 × 10^5^	*Acinetobacter lwoffii*	20
	4.0 ± 0.2 × 10^5^	*Pseudomonas luteola*	15
Outdoor 2 (Bayan)	6.0 ± 0.2 × 10^5^	*Hafnia alvei*	40
	4.0 ± 0.2 × 10^5^	*Acinetobacter lwoffii*	30
	2.0 ± 0.1 × 10^5^	*Pantoea agglomerans*	20
	1.0 ± 0.2 × 10^5^	*Klebsiella ozaenae*	10

### Discussion

The quantitative comparisons between bacterial populations showed a significant quantity and great diversity of bacteria carried by indoor ants compared with outdoor ones. Ant infestations are therefore likely to increase the rate of bacterial dissemination inside human premises and cause infectious and non-infectious adverse health in residential spaces and workplaces at any time [21]. Several studies have mentioned the possible sources for indoor bacteria that often come from human shed skins and natural residues since people spend more than 90% of their time in these indoor environments [21, 22].

The isolation of *E. coli* species in this study from indoor ant samples is of great concern. This bacterium is one of the most commonly examined Gram-negative bacteria in microbiology. Gram-negative bacteria are known to cause sick house building syndrome and cause illness due to the production of endotoxins [23]. Nordell et al. [24] reported that high exposure to endotoxins is often associated with nausea and diarrhea. Though it is well known that it inhabits the human bowel as part of normal microbiota, some strains can cause a chronic diarrhea and sever intestinal infections [25]. Additionally, *E. coli* is the most common etiologic agent of urinary tract infections that may results in cystitis or ends in life threatening sepsis [26]. Another important genus identified by this study were the *Salmonella* which are facultative intracellular pathogens and are recognized as the major causative agents of gastroenteritis and salmonellosis [27].

The analysis of bacterial colony counts of indoor ant samples from Bayan revealed one antibiotic-resistant bacterium *Serratia marcescens* that causes a wide range of infection such as, urinary and respiratory infections, endocarditis, osteomyelitis, septicemia, wound infections, eye infections, and meningitis which formed 30% of the sample. Some of the other species identified, such as, *Shigella, Proteus, Klebsiella, Enterobacter, Citrobacter, Salmonella* and *Yersinia* are also of medical significance as they can cause severe diarrhea and abdominal distress [28]. *Klebsiella pneumoniae*, and *Proteus mirabilis* were also isolated from the ants’ body. They are normal commensal microflora but they may cause opportunistic infection such as hemorrhagic colitis, urinary infection and pneumonia [29–31].

There are several reasons for the augmented high percentage frequency of occurrence in indoor samples, the most important one is the minimal use of disinfection procedures against this insect because people are unaware of the health hazards associated with ants. In addition, dust accumulation coupled with high humidity may lead to the proliferation of microorganisms capable of surviving the prevailing conditions [24]. Indoor air bacteria often originate from outdoors carried in the air in addition to anthropogenic sources such as residents’ activities [21, 32]. Poor aeriation elevates the survival rate of airborne organisms [33]. Eventually, populations of airborne bacteria become high enough to pose a major issue in indoor environments, in particular domestic places and communal areas [20].

The burden of infectious diseases has worsened with the emergence of antimicrobial bacterial resistance, in particular those associated with ants [34]. The current finding highlights the pathogenicity of *Monomorium* spp. ant as it carries, relative to its small body, a wide diversity of pathogenic bacterial species via mechanical means. For instance, the most common organism obtained indoors and outdoors was *Acinetobacter lwoffii*. This bacterium causes serious and sometimes life-threatening infections such as gastrointestinal and urinary tract infections, pneumonia and meningitis [35]. In addition, it is able to survive dry conditions, low pH, and a wide range of temperature levels [36], and is also recognized as a common causative agent of various human diseases such as catheter-associated infections in immunocompromised patients [35, 37].

The comparative analysis showed variability in the integument bacterial composition among the analyzed districts. The studied districts, Bayan and Al-Rowda, differed with respect to location and each is characterized by specific meteorological parameters such as relative humidity. Bayan district is in close proximity to the sea side and is often characterized by high relative humidity compared with Al-Rowda. At high humidity people tend to modify the air temperature of their homes by using ventilators and air conditioning. Indoor bacterial frequency is usually associated with air conditioning filters that facilitate the growth of specific microorganisms [23]. This might be due to the relative humidity, air quality and geographical characteristics. There are other contributing factors that were ascribed to the presence of specific bacteria including, temperature, ventilation strategy, rainfall and building design [38]. Bowers et al. [39] reported that the diversity and composition of the airborne bacterial communities varied across sites and over time. Our data suggest the contribution of composite factors in defining the potential microbial community dynamics including weather conditions and the available microbial sources. Aydogdu et al. [40] and Muhammad et al. [20] noticed that poor ventilation, crowded conditions and increase in the number of air conditioning units inside buildings could facilitate the dispersal and the survival rates of airborne microorganisms that may intensify the probability of air borne infection. Previous studies reported that the occupants’ socioeconomic status, together with the level of hygienic practices may have significant impacts on air pathogens [21, 22, 41].

*Monomorium* ants, therefore, are a dangerous vector contributing to the spread of disease-causing bacteria. Concern must be focused on food storage hygiene, which is regarded as the main source of uncontrollable re-infestation. Homeowners must consider adopting preventive pest management through licensed professionals.

## Limitations

There were some difficulties at the beginning of ant collecting due to their fast movement. Collecting the ante by brushing or direct touching was not allowed at that stage in order to attain reliable results. This was solved by doing several trials before collecting the project sample.
